# Examination of Current Treatments and Symptom Management Strategies Used by Patients With Mal De Debarquement Syndrome

**DOI:** 10.3389/fneur.2018.00943

**Published:** 2018-11-12

**Authors:** Josephine M. Canceri, Rachael Brown, Shaun R. Watson, Cherylea J. Browne

**Affiliations:** ^1^School of Science and Health, Western Sydney University, Sydney, NSW, Australia; ^2^School of Medicine, Western Sydney University, Sydney, NSW, Australia; ^3^Prince of Wales Private Hospital, Sydney, NSW, Australia; ^4^Translational Neuroscience Facility, School of Medical Sciences, UNSW Sydney, Sydney, NSW, Australia

**Keywords:** Mal de Debarquement Syndrome, MdDS, vestibular, neuro-otology, treatment, management strategies

## Abstract

**Introduction:** Mal de Debarquement Syndrome (MdDS) is a neurological disorder which affects the vestibular system pathways, manifesting as a constant sensation of movement in the form of rocking, bobbing, or swaying. The mechanism of MdDS is poorly understood and there is a lack of awareness amongst medical professionals about the condition. This study aimed to examine treatments and symptom management strategies used by MdDS patients and evaluate their self-reported effectiveness.

**Method:** Motion-Triggered and Spontaneous/Other onset MdDS patients responded to a set of comprehensive questions as a retrospective survey regarding epidemiological details, diagnostic procedures, onset, and symptom triggers, hormonal influences as well as treatments and symptom management strategies used to reduce symptoms. The Motion-Triggered questionnaire was made available through Survey Monkey and the Spontaneous/Other Onset questionnaire through Qualtrics. The link for each questionnaire was made available on online MdDS support groups and on various research websites. Descriptive statistics were used for epidemiological data and Pearson's Chi Square tests were used for comparisons between and within both subtype groups.

**Results:** A total of 370 patients participated in the surveys, with 287 valid responses collected for the section regarding treatment and symptom management strategies. The success of the treatments and symptom management strategies did not vary between subtypes Benzodiazepines/Antidepressants were reported as being most beneficial in reducing symptoms in both groups.

**Conclusion:** This was the first attempt to evaluate the reported success of treatments and symptom management strategies in MdDS patients by assessing the patients' perceived helpfulness. The treatments and symptom management strategies reported to be the most helpful in managing and/or reducing symptoms are proposed to be effective due to their stress-reducing capacities. We hope this study will broaden MdDS awareness and that this study will increase patient knowledge regarding treatments and symptom management strategies that other patients found helpful.

## Introduction

Mal de Debarquement Syndrome (MdDS) is a neurological disorder which affects the vestibular system, manifesting as a constant sensation of movement in the form of rocking, bobbing, or swaying (1). The condition is also characterised by fatigue, cognitive impairment, and hypersensitivity to sensory stimuli (2, 3). The development of the disorder in most cases is considered to be a consequence of exposure to passive motion, such as sea travel or driving (2, 4–6), hence these patients are referred to as Motion-Triggered (MT). However, recent research has recognised that patients can report the same symptoms without being exposed to passive motion. Instead, their symptoms occurred spontaneously or following emotionally or physically stressful events, such as surgery, child birth, or during episodes of extreme hormonal change (2, 6). Thus, non-motion related MdDS is unofficially termed Spontaneous or Other-onset MdDS (SO) (2, 6–8). As stated, MT and SO patients are symptomatically comparable, experiencing chronic unrelenting movement (2). However, the symptomatic feature segregating both subtypes of MdDS from other well-known vestibular conditions is that the sensation of phantom motion can be alleviated, to differing degrees, through the re-exposure to passive motion (2, 4, 8, 9).

While the last decade has seen a significant increase in the awareness and clinical investigation of MdDS, knowledge of the condition among medical professionals is still very limited (8, 10). Consequently, the incidence of misdiagnosis and self-diagnosis amongst MdDS patients is high (8), leading to often misdirected and thus ineffective treatments and symptom management strategies. A treatment currently used for reducing MdDS symptoms is the Vestibular Ocular Reflex (VOR) Protocol developed by Dai and colleagues (1, 3). This technique is based on the hypothesis that MdDS is the result of a maladaptation of the VOR. It is proposed that through the use of optokinetic visual stimuli, the VOR can be recalibrated. For additional details see Dai et al. (1). This technique, according to a recent publication, appears to be more successful on MT patients compared to SO patients (3). Currently, hundreds of patients have been treated with the VOR protocol, however research continues into how this protocol can be better refined for MT patients and adapted for SO patients.

Hypothesised to be a disorder of neuroplasticity (11–13), MdDS patients show increased functional connectivity in spatial processing areas and reduced metabolic activity within homologous regions of the frontal, temporal, and parietal lobes (4). Due to these well-documented neural connectivity changes identified by Cha et al. (4), repetitive Transcranial Magnetic Stimulation (rTMS) has been proposed as a promising therapeutic treatment. Although its protocol is currently under development, trials have been conducted by various researchers (14–18), which demonstrate significant potential. To date, a specific rTMS treatment protocol is not currently available for MdDS patients.

As a first course of action, health professionals commonly prescribe benzodiazepines or antidepressants (12, 19) to MdDS patients. In many cases patients are misdiagnosed with mental health issues. Whilst MdDS is not considered a psychological condition by experts and the MdDS community, it has been proposed that mental health issues arise in MdDS patients due to the lack of understanding of the condition by medical professionals, a lack of treatment options, and the intensity and intrusiveness of the symptoms (6).

To date, there have been no investigations evaluating the subjective success of different treatments and symptom management strategies used by those with MdDS. To address this, a comprehensive questionnaire was developed for each onset group, containing questions about epidemiology, diagnosis, onset and symptom triggers as well as hormonal influences (6, 7). The questionnaires also inquired after the treatments and symptom management strategies trialled by patients, and the perceived helpfulness of these therapies in reducing their symptoms. This study aims: to determine the most commonly trialled treatments and symptom management strategies used by both subtypes of MdDS patients; to identify which of these are considered the most helpful in managing or reducing symptoms; and to identify if there are any differences between the two groups regarding which treatments and symptom management strategies are perceived as most beneficial.

## Methodology

### Study population and recruitment

Patients diagnosed by specialists, and those believing to suffer from MdDS (also referred to as self-diagnosed patients) were recruited for the study. Considering the significant misdiagnosis of MdDS, an online questionnaire was developed for both MdDS subtypes to facilitate a global recruitment of as many MdDS patients as possible. The online format allowed for greater accessibility of patients to the study and targeted online resources which are commonly frequented and used by MdDS patients, including the main MdDS support groups: MdDS Australia Facebook Support Group, MdDS UK Facebook Support Group, website of Mount Sinai Hospital, Western Sydney University MdDS Research Group Facebook page, website and Facebook of Vestibular Disorders Association, website and Facebook of Whirled Foundation, and the REACT Community Facebook. MdDS patients were also recruited through the Department of Otorhinolaryngology at the University Hospital of Antwerp, Belgium.

### Inclusion and exclusion criteria

Inclusion criteria: As per the criteria proposed by Mucci et al. (6), built upon guidelines developed by Van Ombergen et al. (20), patients reporting sensations of self-motion (rocking, swaying and bobbing) for longer than 1 month, where the symptoms could not be explained by another diagnosis. Patients reporting MdDS symptoms after exposure to passive motion, most frequently a boat trip, or travel over air or land were denoted as the *Motion-Triggered group (MT group)*. Patients reporting similar symptoms without a clear motion event or any obvious cause were allocated to the *Spontaneous Onset group*. Patients reporting the initial symptoms after a strong emotional or stressful event (e.g., child birth, concussion, physical trauma, surgery, etc.) were defined as the *Other Onset group*. Both “*Spontaneous”* and “*Other”* onset MdDS patients were unified in one group, termed the *SO group*. Self-diagnosed respondents were also included in the survey. Exclusion criteria: Patients who were <18 years old or who did not meet the criteria proposed by Mucci et al. (6) and Van Ombergen et al. (20) for diagnosis of MdDS (2016); principally if respondents indicated that they did not experience temporary relief of symptoms when re-exposed to any form of passive motion (e.g., driving/riding in a car) by responding “No” to the question “Do you feel better or normal when you are riding in a car?.” Data from each self-diagnosed respondent was considered on an individual basis to ascertain whether they were indeed suffering from MdDS by identifying if they answered “No” to this question. If this was the case, their data was excluded from the study. Exclusion of patient data using this criterion resulted in the removal of 10 SO and 2 MT responses, completed by authors JMC and CJB.

### Questionnaires

The questionnaires were approved by the Western Sydney University Human Ethics Committee (H11962). Both questionnaires were available in English only. Questions were created to collect basic epidemiological data concerning this patient group and also to inquire after patient diagnostic, symptom and treatment experiences which remained unaddressed in literature. The questionnaire design was edited and verified by clinical neurology specialists with a great understanding of MdDS and significant experience in MdDS diagnosis. It was also verified by MdDS experts and researchers. The MT questionnaire (51 questions) was distributed using Survey Monkey (SVMK, Inc.) and the SO questionnaire (85 questions) was distributed using Qualtrics (Qualtrics, LLC) following the closure of the MT questionnaire. More questions were made available to the SO group, as the respondents were redirected to one of two specific categories: (1) Spontaneous, and (2) Other, according to their onset. Additionally, more extensive questions about hormonal profiles were included as the questionnaire was developed following the MT questionnaire and therefore allowed for the incorporation of improvements. However, in this manuscript, only the treatment and symptom management strategies questions are analysed, and these questions were the same across both questionnaires (questions available as Supplementary Material). The questions were divided into separate categories for both surveys: epidemiology (demographic details), diagnosis (i.e., who made the initial diagnosis, time frame before receiving the diagnosis, number of appointments), onset triggers (potential triggers related to the onset: events, hormonal fluctuations, medications, stress), symptom triggers (i.e., symptom fluctuation, susceptibility to visual inputs), hormonal influences, and current treatments and symptom management strategies. Though different survey platforms were used for each questionnaire, the flow of questions was consistent. Regarding the questions examined in this manuscript, respondents were asked to identify which treatments and symptom management strategies they had and had not tried in multiple choice format, and were asked to indicate which they considered the most helpful in managing or reducing their symptoms. Respondents were also given the opportunity to add open-ended comments to identify specific medications, remedies or supplements they were or had used to manage symptoms, and to add any additional information they felt was relevant regarding treatments and symptom management (See Supplementary Material). This manuscript focuses on the current treatments and symptom management strategies trialled by respondents and their relative success in reducing MdDS symptoms.

### Statistical analysis

Statistical analysis was performed with SPSS version 24 (IBM Corp). Descriptive statistics were used for epidemiological data and Pearson's Chi Square tests (*p* = 0.05 significance level) were used for comparisons between and within MT and SO groups. Fisher's Exact Test in comparisons which had less than five observations.

## Results

### Epidemiology

Table 1 outlines the epidemiological data collected in this study. A total of 370 responses were collected, with 266 (71.9%) MT and 104 (28.1%) SO participants. Respondents were located across USA, Europe, and Australia, Asia and South America.

**Table 1:** A–G: Demographics, diagnostic experience and cause of MdDS onset of all respondents (*n* = 370) within the Motion-triggered (MT) and Spontaneous/Other (SO) onset group, presented as a percentage of the group and raw number of total responses for each subtype.

**A-Age^a^**	**MT *n* = 266**	**SO *n* = 104**
Mean	48.8 (SD 11.4)	48.9 (SD 13.5)
Total number of respondents that answered this question (%)	99.6% (265)	96.2% (100)
**B-Gender with a missing value category**^a^	**MT** ***n*** = **266**	**SO** ***n*** = **104**
Female (%)	91.0% (242)	88.5% (92)
Male (%)	6.8% (18)	6.7% (7)
Total number of respondents that answered this question (%)	97.7% (260)	95.2% (99)
**C-Location**^a^	**MT** ***n*** = **266**	**SO** ***n*** = **104**
North America	50.9% (135)	51.0% (53)
Europe	25.7% (68)	24.0% (25)
Australia	21.9% (58)	22.1% (23)
Asia	0.8% (2)	1.0% (1)
South America	0.8% (2)	1.9% (2)
Total number of respondents that answered this question (%)	99.6% (265)	100% (104)
**D-Initial diagnosis**^b^	**MT** ***n*** = **266**	**SO** ***n*** = **104**
Self-diagnosed	125 (47%)	33 (35.9)
Otolaryngologist	61 (22.9%)	19 (20.7%)
Neurologist	42 (15.8%)	25 (27.2%)
Health care professionals (physiotherapists, chiropractors, physical therapists, nurses)	23 (8.6%)	15 (16.3%)
General physician (GP)	15 (5.6%)	0 (0%)
Total number of respondents that answered this question (%)	266 (100%)	92 (88.5%)
**E-Number of appointments**^b^	**MT** ***n*** = **266**	**SO** ***n*** = **104**
1	26 (17%)	5 (6.7%)
2–5	68 (44.4%)	24 (32%)
6–10	33 (21.6%)	23 (30.7%)
10–20	17 (11.1%)	12 (16%)
20 to 40	8 (5.2%)	10 (13.3%)
40+	1 (0.7%)	1 (1.3%)
Total number of respondents that answered this question (%)	153 (57.1%)	75 (72.1%)
**F-Cause of MdDS onset- MT subtype**^a^^*^	**MT** ***n*** = **266**
Cruise	162 (60.9%)
Flight	50 (18.8%)
Combination of vehicles (e.g., flight and car; boat and car, etc.)	33 (12.4%)
Train	6 (2.3%)
Car	8 (3%)
Bus	2 (0.8%)
Simulator (virtual reality)	5 (1.9%)
Total number of respondents that answered this question (%)	266 (100%)
**G-Cause of MdDS onset- SO subtype**^a^^*^		**SO** ***n*** = **104**
Stress (psychological, physical)		10 (32.3%)
Strong emotion		5 (16.1%)
As a result of previous vestibular disorder		3 (9.7%)
Physical trauma (e.g., concussion)		7 (22.5%)
Virus		2 (6.5%)
Child birth/pregnancy + hormonal imbalances		3 (9.7%)
Spontaneously (unable to recall a specific event)		1 (3.2%)
Total number of respondents that answered this question (%)		21 (29.08%)

(A) Mean age of MT and SO respondents; (B) Gender distribution within both MT and SO subtypes, (C) Patient location of MT and SO respondents, (D) Initial diagnosis of MT and SO respondents, (E) Number of appointments attended in search for MdDS diagnosis. (F) Triggers for MdDS onset of MT respondents and (G) Triggers for MdDS onset of SO respondents.

a Adapted from Mucci et al. (6).

b Adapted from Mucci et al. (7).

* *Categories presented in the table differ to those offered in the original questionnaires (see Supplementary Data). This refinement of categories was completed due to the variety and great number of answers provided by respondents in order to ensure presentation of the most relevant data. See Mucci et al. (6) for more information*.

Two hundred and eighty-seven responses were collected in the section regarding treatment and symptom management strategies. Within this patient pool, the mean age for respondents within the MT group was 49.9 years (SD 11.4) and 49.8 years (SD 13.0) for respondents within the SO group. 238 (82.9%) were the MT patients, and 49 (17.1%) being of the SO group. A female predominance was observed in both groups, with 222 female respondents (93.3%) in the MT group and 42 (85.7%) in the SO group. In the MT group, 207 (87.0%) were officially diagnosed and 31 (13%) were self-diagnosed. In the SO group, 39 (79.6%) were officially diagnosed and 10 (20.4%) self-diagnosed.

### Trial rate and benefit rate

Respondents were required to identify treatments or symptom management strategies they had used to reduce or manage their MdDS symptoms, and to indicate which of these was the most helpful. On average, MT respondents tried 3.9 different treatments/symptom management strategies, and SO respondents tried 4.5.

**Figure 1:**
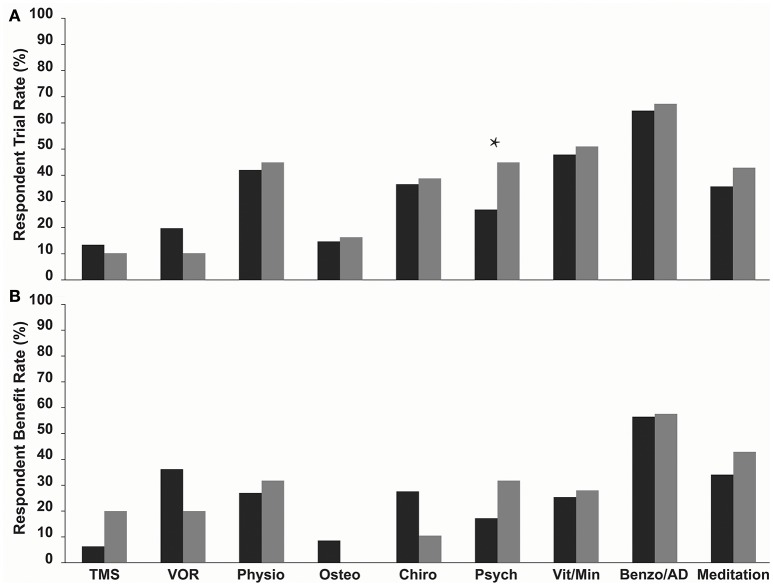
**(A)** Trial rate and **(B)** Reported benefit rate (a reduction or relief of symptoms following treatment) of various current treatments and symptom management strategies amongst MT (dark grey bars) and SO (light grey bars) MdDS respondents. Benzodiazepines/Antidepressants were the most commonly trialled in both groups, and SO respondents on average trialled more treatments and symptom management strategies compared to MT respondents. Benzodiazepines/Antidepressants had the highest benefit rate in both groups. Benefit rates between the two groups did not vary significantly. ^*^p < 0.05. TMS, Transcranial Magnetic Stimulation; VOR, Vestibular Ocular Reflex; Physio, Physiotherapy; Osteo, Osteotherapy; Chiro, Chiropractics; Psych, Psychology; Vita/Min, Vitamins/Minerals; Benzo/AD, Benzodiazepines/Antidepressants.

Benzodiazepine/Antidepressant use was the most commonly trialled treatment in both MT and SO groups, 64.7 and 67.3%, respectively, (Figure 1A, Table 2), followed by vitamin/mineral supplementation (47.9% and 51.0%). TMS and Osteopathy were the least trialled treatments/symptom management strategies in the MT group (13.4 and 14.7%, respectively) and TMS and the VOR protocol in the SO group (both 10.2%) (Table 2). There was a significant difference in the trial rate between MT and SO groups regarding psychology (*p* = 0.012), where a higher percentage of SO respondents tried psychology.

**Table 2:** Trial rate and reported benefit rate (a reduction or relief of symptoms following treatment) for various current treatments and symptom management strategies used by MT and SO respondents to reduce their MdDS symptoms.

	**Total that tried treatment/symptom management strategy**		***p-*value**	**Benefit rate**		***p-*value ^a^(Fisher's Exact Test)**
	**MT (*n* = 238)**	**SO (*n* = 49)**		**MT**	**SO**
TMS	32 (13.4%)	5 (10.2%)	0.538	6.3%	20.0%	0.362^a^
VOR PROTOCOL	47 (19.7%)	5 (10.2%)	0.114	36.2%	20.0%	0.648^a^
PHYSIO	100 (42.0%)	22 (44.9%)	0.710	27.0%	31.8%	0.648
OSTEO	35 (14.7%)	8 (16.3%)	0.772	8.6%	0.0%	1.000^a^
CHIRO	87 (36.6%)	19 (38.8%)	0.769	27.6%	10.5%	0.148^a^
PSYCH	64 (26.9%)	22 (44.9%)	**0.012**	17.2%	31.8%	0.146
VITA/MIN	114 (47.9%)	25 (51.0%)	0.691	25.4%	28.0%	0.791
BENZO/AD	154 (64.7%)	33 (67.3%)	0.724	56.5%	57.6%	0.909
MEDITATION	85 (35.7%)	21 (42.9%)	0.345	34.1%	42.9%	0.455

Pearson's Chi Square Analysis was used and Fisher's Exact Test

(^a^) *was used when any cell of the 2 × 2 table has less than five observations. Significant p-values are in bold text. TMS, Transcranial Magnetic Stimulation; VOR, Vestibular Ocular Reflex; Physio, Physiotherapy; Osteo, Osteotherapy; Chiro, Chiropractics; Psych, Psychology; Vita/Min, Vitamins/Minerals; Benzo/AD, Benzodiazepines/Antidepressants*.

Respondents were asked to indicate which of the trialled treatments and symptom management strategies was the most helpful in reducing symptoms—described here as the “Benefit Rate” (Table 2 and Figure 1B). Benzodiazepine/Antidepressant medication was reported as the most helpful for reducing and/or managing symptoms in both groups; 56.5% for the MT group, where they were significantly rated as the most helpful compared to all other treatments (*p*-values for each comparison were <0.015), and 57.6% for the SO group, where they were significantly rated as the most helpful compared to Osteotherapy (*p* = 0.039) and Chiropractics (*p* = 0.021). Following this was the VOR protocol for MT group (36.2%), then meditation (34.1%), whilst for the SO group it was meditation (42.9%) equally followed by psychology and physiotherapy (31.8%). When comparing the two groups in regards to which of the trialled treatments and symptom management strategies was the most helpful, there were no significant differences observed (Table 2 and Figure 1B).

### Open-ended comments

Ninety-three respondents provided 190 open-ended comments regarding treatments or symptom management strategies they had trialled (that were not listed in the questionnaire) and their perceived helpfulness of that particular treatment/symptom management strategy. Across the 190 comments, 36 various treatments and symptom management strategies were identified, the four most common being *Diet Modification, Magnesium, Vitamin D*, and *Light Exercise*—the remaining 33 are not included in Table 3 as they were only trialled by a small number of respondents (<5% mentions). Diet modification was the most commonly mentioned with 15.8% of respondents indicating that it was useful in reducing their symptoms, and 1.8% indicating that diet modifications did not help. This was followed by Magnesium supplementation which was identified in 14.9% of the comments, then Vitamin D supplementation and light exercise, including walking, Pilates, yoga, and swimming, both at 7.7%, with a 0.5% of comments indicating that light exercise was not successful in reducing symptoms (Table 3).

**Table 3:** Four main categories observed in open-ended comments from MT and SO respondents (combined) regarding “Helpful” treatments/symptom management strategies expressed as raw numbers and percentages.

	**Helpful**	**Unhelpful**
Diet modification	35 (15.8%)	4 (1.8%)
Magnesium	33 (14.9%)	0 (0.0%)
Vitamin D	17 (7.7%)	0 (0.0%)
Light exercise	17 (7.7%)	1 (0.5%)

## Discussion

Investigation into potential treatments for clinical mediation of uncommon disorders such as MdDS can prove difficult, particularly when awareness amongst medical professionals remains limited (2, 6, 10). In response to this need, two online questionnaires were created to collect data from MdDS patients around the world. In total, 370 respondents completed either the MT or SO questionnaire; 238 MT and 49 SO specifically completed the questions regarding treatments and symptom management strategies that they felt were helpful in reducing their symptoms. The current study is the largest in terms of MdDS respondents recruited to date, and is the only survey that has collected information about the patients' experience with treatments and symptom management strategies which are perceived to be beneficial in reducing symptoms or helping patients manage their symptoms.

Respondents from the MT and SO groups showed similar epidemiological results. The average age was 49 years old for both groups, with a strong female predominance. These results are comparable to a mean age of 45 years reported in other studies (3, 9, 20, 21).

According to Hain and Cherchi (19), a MdDS diagnosis may be given after 1 month from onset of continued Mal de Debarquement symptoms, after which the common strategy is to make the patient comfortable in the anticipation that symptoms will dissipate independently over the first 6 months (19). During this time, Benzodiazepine or Antidepressant medications are commonly prescribed (12, 19). If symptoms have not improved, other interventions and potential treatments should be explored. Hain and Cherchi (19) suggest that rTMS treatments (13, 15, 22) as well as visual stimulus-based habituation through the VOR protocol (1) seem the most promising treatments for addressing MdDS symptoms.

Due to a lack of understanding of the underlying mechanisms of MdDS, a viable treatment is not currently available to MdDS patients of both subtypes. In addition to this, the lack of awareness amongst medical professionals commonly leads to misdiagnoses (6, 12, 23) and therefore treatment plans not always specific to the condition. This manuscript demonstrates the broad range of treatments and symptom management strategies that MdDS patients use in order to reduce symptoms in an attempt to manage their symptoms. As MdDS symptoms are intrusive and even debilitating for some (6, 10) Pearce and Daws, unpublished), it is understandable that patients often trial many different treatments and symptom management strategies as indicated by this study where, on average, MT respondents tried 3.9 different treatments/symptom management strategies and SO respondents tried 4.5. As SO patients are more commonly misdiagnosed (6), this could suggest that SO patients engage in a higher rate of misguided treatments and symptom management strategies that are not specific for managing MdDS. This is further supported by our data in which respondents of the SO group had trialled the majority of treatments and symptom management strategies at a higher rate than those within the MT group.

There were no significant differences between the MT and SO groups regarding the perceived benefit of treatments and symptom management strategies trialled. This suggests that MT and SO may have a similar underlying mechanism, with the only difference being the onset cause. Importantly, the only treatment that showed a dissimilar benefit rate (a reduction or relief of symptoms) between the two groups was the VOR protocol, though this was not statistically significant. This supports previously published research, which showed that the VOR protocol produces higher success rates in those with MT MdDS than those with SO MdDS (1, 3, 24). The VOR protocol is based on the theory that MdDS patients experience difficulty in readjusting to new stable situations as information is retained for gaze stabilisation during a (preceding) context of motion. This may explain the discrepancy in the effectiveness of the protocol between MT and SO patients, as SO individuals may not have been exposed to the same stimuli which can induce the aberrant nystagmus that often manifests from maladaptation of the VOR (3, 6).

Across both groups, it was clear that Benzodiazepine/Antidepressant medications were reported to be the most trialled, and felt to be the most successful in reducing symptoms. These medications are often a common avenue of medical treatment for MdDS patients (12, 19, 25). The majority of respondents who reported trials of Benzodiazepine/Antidepressant medications indicated that they were the most helpful in reducing symptoms for both MT (56.5%) and SO (57.6%) groups. This affirms previous literature findings which described the overall success of such medications in addressing MdDS symptoms (9, 13, 21, 26–28). Though the underlying mechanisms are unknown, the psychological implications of MdDS have proven quite severe for patients, with depression and anxiety common features of the disorder as a consequence of and not a cause for the condition (1, 6, 21). A recent publication by Mucci and colleagues (6) demonstrated that these psychological symptoms are highly likely to manifest as part of MdDS symptomology and contribute to heightened symptoms. This is understandable considering respondents indicated that stress was a major trigger for their symptoms and that both anxiety and depression are common consequences of prolonged or repetitive stress (29). Previous epidemiological studies found MdDS participants had a mean depression score of 40.79 and, according to Radloff's criteria, were deemed to be suffering from major depression (10). This is understandable considering the intrusiveness of the condition into most facets of an individual's life, with survey analysis reflecting a mean total illness invasiveness factor of 44.42 (10), coupled with the stigma of having a disorder largely invalidated by the medical community (2, 5, 6, 10, 23, 27).

Hain et al. (9) found that benzodiazepines (specifically clonazepam and diazepam) to be amongst the most helpful medications for MdDS patients. Parker and Jennings (26), as well as Saha and Fife (13), similarly identified clonazepam as the most commonly helpful, preferentially due to its longer half-life (13). Benzodiazepines are common pharmacological treatment for anxiety disorders (30–32). Such anxiolytic drugs act predominately upon gamma-aminobutyric acid (GABA)-A receptors (29) by binding via a modulatory binding site, (33) increasing the sensitivity to and thus activity of gamma-aminobutyric acid (GABA). As GABA is an inhibitory neurotransmitter, it facilitates hyperpolorisation which in turn reduces the excitability of neurons (34). This induces a calming effect on the body (29, 35) and subsequently producing the anticonvulsant, musculorelaxant, anxiolytic, and sedative effects which characterise these drugs (34). As neuroimaging studies of anxiety patients have reflected reductions in GABA levels and GABA-A benzodiazepine receptor binding (36), this substantiates the anxiolytic results achieved through benzodiazepine administration and may suggest why MdDS patients, who experience high prevalence of anxiety symptoms (6), reported high rates of benefit as demonstrated in this study. Benzodiazepines have also been found to exert neuroendocrine effects, which are hypothesised to mediate the stress-induced hyperactivation of the hypothalamic-pituitary axis (HPA) which occurs in anxiety disorders (36). This modulated activity is believed to occur at the hypothalamic and/or suprahypothalamic level by suppressing the production of corticotrophin-releasing hormone (37). Such overstimulation is demonstrated by elevated peripheral levels of adrenocorticotrophin-releasing hormone (ACTH) and cortisol, as well as reduced ACTH response to corticotrophin-releasing hormone (36, 38, 39). Thus, it is hypothesised that through benzodiazepine administration, stress-induced pathways involved in the pathology of anxiety disorders can be alleviated by downregulating the activity of the HPA axis and thus the sympathetic nervous system (37, 38), thereby providing relief of anxiety symptoms. However, benzodiazepines are similarly unlikely to provide on-going improvement of symptoms (13) as the acute inhibitory effect on the HPA is found to dissipate with long-term administration (40) through down-regulation of receptor function (29).

Combined treatment regimens involving benzodiazepine and antidepressants medications are commonly implemented (39), as anxiety disorders are often found to be precursor to or comorbid with depression (41). Regarding depression, a “corticosteroid receptor hypothesis” (42) postulates that brain and potentially corticotrophin glucocorticoid receptor expression or functionality is defective in depressed patients (40); this impaired corticotrophin glucocorticoid receptor-mediated feedback inhibition may potentially explain the elevated baseline HPA activity also observed in depression patients (43). This hypothesis continues by suggesting that this receptor deficit can be mediated and reversed through appropriate medication of antidepressants. Whilst the mechanism by which these medications reduced HPA activity remains unclear, it has been suggested that they may involve: (i) monoamine effects on corticosteroid receptor expression or activity, (ii) changes in glucocorticoid access to the brain, and (iii) changes in downstream signalling pathways engaged by glucocorticoids, as described by Jacobson (40). Such antidepressants include tricyclic antidepressants and selective serotonin reuptake inhibitors (SSRI). Whilst both have been found to be effective, SSRI are encouraged in preference due to their greater safety and tolerability (41) and were commonly noted by some SO and MT respondents. The mechanism of action for SSRIs involves inhibition of serotonin transporter reuptake protein (44). This increases the serotonin concentration within the synaptic cleft by inhibiting the activity of serotonin reuptake (44). The efficacy of SSRI medications for MdDS patients could be attributed to the effect of the drug within the hippocampus. In rodents, serotonin is found to reduce activity of CA-1 cells, thereby down-regulating hippocampal output, though the exact mechanism by which this occurs is determined by the receptor subtype (45). Additionally, the SSRI sertraline has been shown to increase hippocampal neurogenesis in humans (46). As the hippocampus is found to be hyperactive within the MdDS brain (4), inhibitory action of SSRIs could be operating to silence the excessive stimuli projected from the region (45). This in turn may lessen MdDS symptom severity. Long-term use however, can downregulate and desensitise the serotonin receptors (45, 47), perhaps explaining the limited effectiveness of long-term use as noted by Parker and Jennings (26). Antidepressants have also been found to heighten hippocampal neurogenesis in animal (48) as well as human models (46). Thus, as atrophy of the hippocampus exists as a hallmark neuroplastic feature of depression (49), it could be argued that the action of antidepressants may regulate connectivity within the hippocampus of MdDS patients by promoting cellular regeneration; which may be compromised as a consequence of developing MdDS-associated depression (6).

Therefore, benzodiazepines and antidepressant medications are proving to be a promising treatment in reducing symptoms for some MdDS patients. This does not appear to be because MdDS has a psychological basis, but rather due to the ability of these medications to mediate emotional distress associated with MdDS, limit physiological responses to stress or potentially challenge part of the neural hyperactivity that is theorised to cause the disorder. However, as highlighted, long-term use of these medications may decrease in efficiency over time due to desensitisation. It is important to note that the high success of this treatment may also be associated with its considerable accessibility. When compared to other treatments which are still being optimised (i.e., rTMS), or have limited availability around the world (i.e., the VOR protocol), these medications provide an affordable and easily accessible treatment option. Thus, the viability of benzodiazepines and antidepressants as a successful treatment strategy must be verified in the context of its ease of access, as well as its mediation of the emotion and psychological symptoms of MdDS development.

Other beneficial symptom management strategies reported in this study to assist the reduction of MdDS symptoms for both MT and SO respondents were psychology and meditation, with higher rates in the SO group. Meditation was the second (for SO group) and third (for MT group) most helpful symptom management strategy reported, and psychology was rated as the third most helpful strategy for the SO group. The benefit observed in our study of psychotherapeutic strategies such as meditation and psychological counselling is likely to go beyond mediating the depressive and anxious symptoms that often impact MdDS patients. In this study, a 17.2% (MT) and 31.8% (SO) benefit rate was reported for psychological counselling, and 34.1% (MT) and 42.9% (SO) for meditation. It is commonly known that anxiety and stress can impair cognitive function, causing dendritic atrophy and reduced spine density, thereby retarding effective functioning (50). One of the focal points of such neuronal deterioration in these psychiatric diseases is the hippocampus (49), which has been implicated in the structural and connectivity alterations that occur in MdDS patients (4). As spatial navigation, a responsibility of the hippocampus, is one of the main cognitive aspects of vestibular compensation that can be sensitive to acute and chronic stress (51), it is understandable that such therapies could therefore produce a psychosomatic effect for MdDS patients and contribute to symptom relief or reduction. Psychological counselling may also aid patients by addressing the stress associated with the condition and the lack of understanding provided generally by the medical community, as well as the subsequent sense of isolation (2, 5, 6, 10, 23, 27). This in turn may help patients accept their condition and develop a more positive outlook, further challenging the symptoms of depression and anxiety associated with the condition.

Vitamin and minerals were shown to be helpful in around a quarter of respondents from both groups (25.4% MT and 28.0% SO). In the open-ended comments data, Magnesium and Vitamin D were commonly mentioned. Magnesium deficiencies have been correlated to anxiety and depression (52–55) and migraine pathogenesis (56, 57), with supplementation reducing the frequency of migraine attacks (56, 58, 59). As migraine is hypothesised to be caused by predisposed hyperactivity of the brain (59), and is often an associated symptom of MdDS (21, 25), the addition of magnesium could be serving to combat hyperactivity within hypermetabolic regions of the MdDS brain, or perhaps reducing stress levels by interacting with various neurotransmitter and stress pathways (60). There is an increasing body of evidence suggesting that vitamin D deficiency can enhance susceptibility to the development of depression (61, 62). Subsequently, supplementation has been found to reduce depressive symptoms (63, 64). Whilst the exact mechanism is unclear, it is believed that the addition of vitamin D acts by reducing the high intracellular levels of calcium ions, which have been found to drive depression (65). Therefore, the relatively common use and reported success of vitamin D by MdDS patients could be attributed to the relief it may provide to the symptoms of depression which are often associated with developing the disorder (6). Vitamin D supplementation has also been shown to reduce the occurrence of migraine (66). Light exercise was another symptom management strategy that was apparent from the open-ended comments, which included walking, yoga, Pilates and other non-stressful exercises. Light exercise is known to have a myriad of benefits in boosting overall health. With specific regards to MdDS, it is known to boost endorphin release that is beneficial to mental health (67–69), decreases the occurrence of migraine (70–72) and aids in vestibular and reflex strengthening (73, 74). MdDS has been hypothesised to be a migraine variant and Ghavami and colleagues (25) conducted a study treating MdDS patients with vestibular migraine treatments. Their MdDS patients responded well to management with a vestibular migraine protocol, which included lifestyle changes (diet modifications), as well as pharmacotherapy. In our data, diet modifications were the top mentioned symptom management strategy in the open-ended comments section. This is opposite to the findings of Cha et al. (21), who found that patients did not find relief from their symptoms by utilising diet modifications. However, it is likely that the reported dietary changes that patients trialled could vary drastically and in many different ways, and therefore the true effectiveness of this technique could only be validated through the trialling of a common diet amongst a patient pool, which is yet to be investigated.

Postural instability is a common feature of MdDS (1, 75). Our data suggests that vestibular and reflex strengthening exercises may benefit MdDS patients. Our results have shown physiotherapy was the top third most trialled treatment for both groups, with a smaller majority trialling chiropractics and osteotherapy; 31% of SO respondents indicated that physiotherapy was helpful in reducing symptoms and 27% and 27.6% of MT respondents indicating that physiotherapy and chiropractics (respectively) were helpful in reducing symptoms. This supports some previous findings (21), but contradicts others (25). Vestibular rehabilitation or vestibular physical therapy (VPT) has become an increasingly common management strategy for persistent dizziness and vertigo disorders (76). Our study has shown that patients experienced reduced symptoms after, to what is assumed to be generalised vestibular and reflex strengthening, and balance exercises. However, specific physiotherapy or chiropractic treatment for those suffering from MdDS is yet to be established. Generalised physiotherapy and chiropractic treatments involve development of exercise protocols, combining physical movements with exposure to a diverse range of sensory stimuli in order to reduce symptoms as well as improve dynamic stability and balance (77). These exercises often intentionally induce a visual-vestibular or somatosensory conflict (76) so that patients learn to adapt to the error signal interpreted by the vestibular system and accommodate the gain via specific coordinated eye and head movements (78). These strategies are classified into: vestibular adaptation, which implements hand-eye coordination exercises in order to recalibrate the VOR (18, 50) in the treatment of gaze instability (76); vestibular habitation, involving movement-focused exercises which expose the patient to provoking stimuli and attempt to achieve desensitisation through repetition of provocative movements (51, 76, 78) in order to reduce position-induced dizziness (even though MdDS patients do not describe their symptoms as dizziness); and vestibular substitution, centring on reprioritising the utilisation of visual and proprioceptive inputs and re-learning movements to limit the inducement of vertigo-like sensations (50, 51, 77). Clinical trials of this format on Benign Paroxysmal Positional Vertigo patients have demonstrated improvements in dizziness and balance as it enables patients to better integrate all somatosensory senses (77). The effectiveness of such physical therapy is likely to be two-fold: psychosomatically, in addressing the psychological element of vestibular disorders in terms of anxiety (76, 78, 79), as patients gain greater confidence with their physical movement and daily activities, seeing as high levels of kinesiophobia often accompany vestibular disorders (51, 76) including MdDS (75), and secondly, due to the finding that MdDS patients, like patients of motion sickness, may rely more so on the reception of their somatosensory system rather than their vestibular system to maintain balance. Thus, VPT reprioritisation of sensory stimuli may be facilitating mitigation of the stimulus conflict which is hypothesised to play a part in the pathogenesis of the condition (13).

The treatments and symptom management strategies discussed previously have shown some success in helping reduce the symptoms of MdDS patients, mainly via their stress-reducing capacities, their association with treating migraine, and their ability to strengthen vestibular reflexes and balance. However, these treatments and symptom management strategies may not have the capacity to “cure” the condition, as they are unlikely to address the neuroplastic adjustments which accompany MdDS as a neurological disorder. However, the lack of understanding regarding the mechanisms by which these treatments and symptom management strategies act on the brain restricts our conclusions, and therefore all deductions are speculative.

Treatments which seek to address MdDS symptomology by inducing beneficial neuroplasticity in order to readjust maladaptive connectivity seen in MdDS patients (4) are the VOR protocol and rTMS treatment. The readaptation of the VOR is the objective of the VOR protocol developed by Dai and colleagues in 2014 (1). In this study, the VOR protocol was not a common treatment that the respondents had tried; SO respondents trialled it less so than the MT respondents. The low trial rate may be attributed to the high cost of the treatment, its limited availability in the world, and the possibility that some patients were cured by the treatment and have subsequently failed to remain actively engaged in MdDS support groups, thereby avoiding recruitment for the questionnaire. Dai's preliminary results, released in 2014, indicated that MdDS symptoms were resolved in 23/24 patients; 6 months later 17 of these 24 patients were cured or had substantially reduced symptoms for a significant period (average 11.6 months) (1). Continuation of these studies found that the 120 MT and 21 SO patients reported an initial improvement rate of 78 and 48% respectively 1-week post-treatment; however, 1-year post-treatment the symptom reduction success rate dropped to 52% for MT and remained at 48% for SO (3). Whilst they found no difference in improvement rate between MT and SO patients 1 year on (3), more recent studies have reported higher success amongst MT patients compared to SO (24). Data from our study reflects a different degree of benefit from the treatment as experienced by respondents; MT patients reported 36.2% success rate whilst the rate for SO patients was 20%. The VOR protocol is designed to provide optokinetic visual stimuli, which is believed to affect the VOR as well as the velocity storage mechanisms of the brain. The vestibular nuclei, inferior olive and cerebellum are the neuronal centres which are proposed to be involved in the disruption of normal VOR (1, 80). Thus, it is theorised that the frequency and amplitude of this synthetic proprioceptive stimuli mutes or phases out this irregular signal which perpetuates the false sensations of motion (4). Therefore, according to Dai and colleagues, it can be stated that this protocol is more appropriate to treat MT patients as their condition had been induced by passive sensations of motion, which are likely to retard the activity of these neuronal centres and establish this abnormal underlying oscillating rhythm. However, SO manifests the same symptom profile without the inducing stimulus and this may explain the limited success amongst these patients. It is also noteworthy that whilst the VOR protocol provided relief to some for their oscillating symptoms, susceptibility to visual and motion stimuli, such as intolerance to fluorescent lights or busy patterns, remained unchanged (3). It is clear that though the VOR is an established treatment available for MdDS patients, there is still potential for refinement and optimisation in the hope of obtaining higher success rates, with long term symptom reduction in both MT and SO patients.

Another treatment that is theoretically promising is rTMS. As previously described, brain activity in MdDS patients has shown to possess an abnormally high resting state of functional connectivity within sensory-processing areas (2, 11). Identification of the excitability and neuroplastic changes which occur in the brains of MdDS patients has led to the proposal of rTMS as a potential treatment for MdDS (2, 27, 75, 81). Currently there is no rTMS protocol available that specifically targets MdDS, thus the respondents in our study that indicated to have trialled rTMS may have done so in a research setting, in the trials conducted by Cha (14, 16), Chen (18), Shou (17, 22), or Pearce (15), or that respondents received rTMS treatments for anxiety and depression. As reported in our data, rTMS was the least trialled treatment in both groups. The benefit rate in the MT group was the lowest across all treatments and symptom management strategies and the third lowest in the SO group. This does not suggest that rTMS is not a viable treatment for MdDS, but highlights the fact that a specific MdDS rTMS protocol for MdDS is not yet available for patients and that the protocol is in its early developmental stages. However, rTMS offers strong potential as a treatment for MdDS in the future. Current research suggests that rTMS for MdDS patients is the most optimal when coupled with electroencephalography neural synchrony and functional conductivity changes as a guideline for the stimulation (22). Pearce et al. (15) conducted rTMS treatment on 13 MT patients with targeted stimulation of the dorsal lateral prefrontal cortex (DLPFC), a region commonly reflecting hypometabolism in MdDS patients (4). Patients reported a reduction in phantom motion symptoms of “rocking” and “bobbing,” and described associated improvement when undertaking previously symptom-provoking activities (such as traveling through crowds). Correspondingly, Shou et al. (22) conducted a similarly structured protocol in which stimulation of the DLPFC produced activity changes in functionally connected regions (visual cortex, supplementary motor areas and PFC), which produced an overall reduction in MdDS symptoms for patients. Like the study undertaken by Cha and colleagues (14), such experimentation has found that stimulation of the DLPFC ipsilateral to side of dominance for writing provides the greatest improvement. Additionally, low frequency stimulation was, on average, linked with symptom worsening (14, 16). Treatment of this brain region may be relevant for MdDS patients as DLPFC hypoactivity has been found to be causally involved in the pathogenesis of depression (82).

Following rTMS of the entorhinal cortex (EC), an improvement of symptoms was reported, which produced downgraded connectivity within the bilateral EC, right inferior parietal lobule and precuneus (18). This is of interest, as the left EC was found to be hypermetabolic in MdDS patients, with increased functional connectivity within posterior spatial processing areas. Subsequently, rTMS research has demonstrated that the longer the duration of MdDS symptoms, the less benefit is derived from the procedure; however sequential days of treatment are highly likely to have produced a more substantial effect in symptom alleviation (16). These findings infer the current theory of MdDS manifesting from the oversynchronisation of brain networks caused by entrainment to the background of low amplitude oscillating stimuli. It can therefore suggest that introduction of rTMS periodic stimuli interferes with this abnormal rhythm of deregulated stimulus (14). The low rate of reported success amongst the respondents in this study is relatively inconclusive, seeing as we have no knowledge of the specific stimulation and technique those patients were subjected to, as well as other variables. It is known that if the target region for rTMS is not accurately directed by navigation tools, the implementation of rTMS can then be considered poor, resulting in a low success rate, and this may be an important factor in the reported benefit rate in the study.

### Study limitations

Access to patients was limited to those active on social media and those who may have visited webpages that promoted our studies. Access to some patients would have also been limited as the survey was only provided in English and was unable to be translated into other languages.

The study was primarily limited by the fact that we do not expect the questionnaire would have been completed by those who have gone into remission, as (we speculate that) these patients would be less active or completely unengaged with MdDS support groups. However, it should be acknowledged that there may be some patients in remission also utilising these support mediums to assist current sufferers. Therefore, we may possibly be missing the data of potential individuals who have been “cured” by treatments highlighted in this study. Accordingly, the study is limited as no response rate can be established as the links were widely available through different online avenues and therefore the level of participation was dictated by the number of individuals who accessed the survey. This study was also limited by the potential for the inaccurate recollection of respondents, as well as their lack of knowledge regarding specific details or the exact category regarding the mechanisms of actions of any prescribed medication. An additional limitation was the absence of a control group; however, this was addressed by conducting a broader literature review to examine the reported effectiveness of relevant medications or supplements according to previously published studies.

We are aware that the number of SO respondents was limited and less than the MT group, but this is likely a reflection of the broader ratio of MdDS patients (6). In addition to this, the definition between “other onsets” and “spontaneous” onsets could have potentially been better clarified to the respondents of the SO survey, who for the first time had to self-define if they had “spontaneous” or “other onset” MdDS. Some respondents in this study were self-diagnosed, however we assumed that many were able to diagnose themselves through resources available on the Internet. Ideally, a larger patient pool where all respondents have received an official MdDS diagnosis would be preferable in future studies.

## Conclusion

Current treatments and symptom management strategies of this unusual vestibular disorder are varied. Benzodiazepine/Antidepressant medication was the most trialled and most beneficial in reducing the symptoms of MdDS of the respondents in this study. In line with previous research, stress-reducing treatments, and symptom management strategies do seem relatively effective in reducing symptoms. However, the symptom relief gained from these methods of treatment is more likely to be associated with the reduction of depressive or anxiety symptoms, and reducing physiological stress responses. Until more is understood about the pathophysiological mechanisms of the disorder, no assured means of treatment can be recommended. Though patient experience is varied and responses to trialled treatment are patient-dependant, the anecdotal evidence collected may provide some guidance to newly diagnosed patients or patients that have exhausted many treatment options. From the findings of this study, it is recommended that patients trial Benzodiazepine/Antidepressant medications as a preliminary treatment. By addressing the depressive or anxiety symptoms associated with MdDS, such medication may help reduce symptoms exacerbated by stress and may also assist patients in accepting their condition with greater positivity and emotional stamina. It remains to be seen whether it might potentially act upon some undiscovered and unhypothesised mechanism to alleviate symptoms in MdDS patients. Such medication could then be coupled with physical therapy in order to strengthen vestibular reflexes and balance. Further research into the refinement and optimisation of the VOR protocol and development of the rTMS protocol, or other neuromodulation techniques, may provide a crucial opportunity for the development of a curative treatment and enhanced understanding of MdDS pathophysiology. Fundamentally, more research is needed to elucidate the underlying mechanisms of MdDS in order to improve treatment techniques and provide optimal patient care and quality of life.

## Data availability

The datasets generated and/or analysed during the current study are not publicly available due to the confidential nature of the data.

## Ethics statement

Ethical approval was provided by the Ethics Committee of the University Hospital Antwerp Belgium (IRB number 15/44/454) and by the Western Sydney University Human Ethics Committee (H11962). Prior to commencement of the questionnaire, each respondent was required to read a Patient Information Sheet which provided all details regarding the study; this document stipulated that completion of the questionnaire served as consent for anonymous responses to be used for research purposes and to be published. Thus, participation and completion of the study served as the informed consent of the respondent. All investigations have been conducted according to the principles expressed in the Declaration of Helsinki.

## Author contributions

JC is the primary author, and performed the literature review, assisted in analysis of the data and wrote the manuscript. RB helped design the survey and assisted in the editing of the final manuscript. SW helped design the survey and assisted in editing the final manuscript. CB is the senior author, and designed the study, performed analysis of the data and assisted in writing and editing the manuscript.

### Conflict of interest statement

The authors declare that the research was conducted in the absence of any commercial or financial relationships that could be construed as a potential conflict of interest.

## Abbreviations

ACTH: adrenocorticotrophin-releasing hormone
DLPFC: dorsal lateral prefrontal cortex
EC: entorhinal cortex
GABA: gamma-aminobutyric acid
HPA: hypothalamic-pituitary axis
MdDS: Mal de Debarquement Syndrome
MT: Motion-triggered MdDS subtype
rTMS: repetitive Transcranial Magnetic Stimulation
SO: Spontaneous/Other onset MdDS subtype
SSRI: selective serotonin reuptake inhibitor
VOR: Vestibulo-ocular reflex
VPT: vestibular physical therapy.
