# Supplementary material for: Examination of Current Treatments and Symptom Management Strategies Used by Patients With Mal De Debarquement Syndrome
Source: Front Neurol. 2018 Nov 12;9:943. doi: 10.3389/fneur.2018.00943 (PMC6240763; doi:10.3389/fneur.2018.00943)
Supplement: Supplementary file 1 [file Data_Sheet_1.pdf]

Supplementary Material

Examination of Current Treatments and Symptom Management Strategies Used by Patients with Mal De Debarquement Syndrome

Josephine M. Canceri*, Rachael Brown, Shaun Watson and Cherylea J. Browne

*** Correspondence:** Josephine Canceri: j.canceri@westernsydney.edu.au

1 Supplementary Data

Questions analysed and discussed in this manuscript

MOTION TRIGGERED (MT) QUESTIONNAIRE

1. BASIC INFORMATION

1.2: Country/State/City:
1.3: Sex:
[ ] Male
[ ] Female
1.4: Date of Birth

2. MdDS DIAGNOSIS

2.1: Who initially diagnosed you with MdDS:

[ ] Neurologist

[ ] Otolaryngologist

[ ] Physiotherapist

[ ] Self-diagnosed

[ ] Other [Free text box]

2.2: Who diagnosed you with MdDS after your initial diagnosis: (e.g. if you were self-diagnosed initially and received an official diagnosis subsequently) and was the specialist/health care professional confident in the diagnosis?

[ ] Neurologist

[ ] Otolaryngologist

[ ] Physiotherapist

[ ] Other [Free text box]

[ ] N/A as initial diagnosis was the only diagnosis

[ ] N/A as self-diagnosed is the only diagnosis

2.4: If you have been diagnosed by a medical professional, provide an estimate of how many medical appointments you attended before your MdDS diagnosis (for example if you were sent to a physiotherapist, radiologist, etc.).

[ ] 1

[ ] 2-5

[ ] 6-10

[ ] 10-20

[ ] 20-40

[ ] 40+

2.5: If you are self-diagnosed, provide an estimate of how many medical appointments you have attended in the quest for an official diagnosis (for example if you were sent to a physiotherapist, radiologist, etc.).

[ ] 1

[ ] 2-5

[ ] 6-10

[ ] 10-20

[ ] 20-40

[ ] 40+

3. MdDS ONSET AND SYMPTOMS

3.1: To the best of your knowledge, what was the motion event that induced your MdDS? Select one answer:

[ ] Short cruise (less than a day)

[ ] Long cruise (more than a day)

[ ] Short airplane flight (<3 hours)

[ ] Long airplane flight (>3 hours)

[ ] Short Train ride (<3 hours)

[ ] Long Train ride (>3 hours)

[ ] Short Car ride (<3 hours)

[ ] Long Car ride (>3 hours)

[ ] Short Bus ride (<3 hours)

[ ] Long Bus ride (>3 hours)

[ ] Short Tram ride (<3 hours)

[ ] Long Tram ride (>3 hours)

[ ] Fairground/theme park ride

[ ] Other [Free text box]

6. SYMPTOM MANAGEMENT AND TREATMENT

6.1: Please indicate which treatments / symptom management strategies that you have and have not tried to reduce or manage your symptoms and indicate which you felt was the most helpful.

[ ] Transcranial magnetic stimulation (TMS)

[ ] Dr Dai’s protocol / VOR protocol

[ ] Allied health – physiotherapy

[ ] Allied health – osteotherapy

[ ] Allied health – chiropractic

[ ] Mental health – psychology

[ ] Vitamin or mineral supplements

[ ] Medication – benzodiazepines/antidepressants

[ ] Meditation

[ ] Other [free text box]

6.2: Is there anything you would like to add about symptom management and treatments for MdDS or any experience that you feel is appropriate to this section?

[free text box]

SPONTANEOUS/OTHER ONSET (SO) QUESTIONNAIRE

1. BASIC INFORMATION

1.2: Country/State/City:

1.3: Sex:

[ ] Male

[ ] Female

1.4: Date of Birth

2. MdDS DIAGNOSIS

2.1: Who initially diagnosed you with MdDS:

[ ] Neurologist

[ ] Otolaryngologist

[ ] Physiotherapist

[ ] Self-diagnosed

[ ] Other [Free text box]

2.2: Who diagnosed you with MdDS after your initial diagnosis: (e.g. if you were self-diagnosed initially and received an official diagnosis subsequently) and was the specialist/health care professional confident in the diagnosis?

[ ] Neurologist

[ ] Otolaryngologist

[ ] Physiotherapist

[ ] Other [Free text box]

[ ] N/A as initial diagnosis was the only diagnosis

[ ] N/A as self-diagnosed is the only diagnosis

2.4: If you have been diagnosed by a medical professional, provide an estimate of how many medical appointments you attended before your MdDS diagnosis (for example if you were sent to a physiotherapist, radiologist, etc.).

[ ] 1

[ ] 2-5

[ ] 6-10

[ ] 10-20

[ ] 20-40

[ ] 40+

2.5: If you are self-diagnosed, provide an estimate of how many medical appointments you have attended in the quest for an official diagnosis (for example if you were sent to a physiotherapist, radiologist, etc.).

[ ] 1

[ ] 2-5

[ ] 6-10

[ ] 10-20

[ ] 20-40

[ ] 40+

3. MdDS ONSET AND SYMPTOMS

3.1: Do you think your MdDS was triggered by an event, which was not motion, for example: trauma, concussion, childbirth, strong emotion (other onset); or did the onset of your MdDS seem to have no obvious cause (spontaneous onset)? Your answer will direct to you specific ‘other’ or ‘spontaneous’ onset questions.

[ ] I think that my MdDS onset was triggered by an event which is not considered passive motion. (‘other’)

[ ] I think that I had a spontaneous MdDS onset, as I cannot recall a specific event. (spontaneous)

FOR SUBJECTS ANSWERING ‘OTHER’:

OTHER event 3.2: To the best of your knowledge, what was the event that induced your MdDS?

[ ] Concussion

[ ] Trauma (physical or psychological)

[ ] Childbirth

[ ] Pregnancy

[ ] Strong Emotion

[ ] Other [Free text box]

OTHER event 3.5: Were you under a lot of stress when symptoms first appeared?

[ ]Yes

[ ] No

[ ] Not sure

FOR SUBJECTS ANSWERING ‘SPONTANEOUS’:

SPONTANEOUS 3.4: Were you under a lot of stress when symptoms first appeared?

[ ]Yes

[ ] No

[ ] Not sure

6. SYMPTOM MANAGEMENT AND TREATMENT

6.1: Please indicate which treatments / symptom management strategies that you have and have not tried to reduce or manage your symptoms and indicate which you felt was the most helpful.

[ ] Transcranial magnetic stimulation (TMS)

[ ] Dr Dai’s protocol / VOR protocol

[ ] Allied health – physiotherapy

[ ] Allied health – osteotherapy

[ ] Allied health – chiropractic

[ ] Mental health – psychology

[ ] Vitamin or mineral supplements

[ ] Medication – benzodiazepines/antidepressants

[ ] Meditation

[ ] Other [free text box]

6.2: Is there anything you would like to add about symptom management and treatments for MdDS or any experience that you feel is appropriate to this section? *

[free text box]

2 Supplementary Figures and Tables

N/A

2.1 Supplementary Figures

N/A
